# PHF2 regulates sarcomeric gene transcription in myogenesis

**DOI:** 10.1371/journal.pone.0301690

**Published:** 2024-05-03

**Authors:** Taku Fukushima, Yuka Hasegawa, Sachi Kuse, Taiju Fujioka, Takeshi Nikawa, Satoru Masubuchi, Iori Sakakibara

**Affiliations:** 1 Department of Physiology, School of Medicine, Aichi Medical University, Nagakute, Aichi, Japan; 2 Department of Nutritional Physiology, Institute of Medical Nutrition, Tokushima University Graduate School, Tokushima, Japan; Fujita Health University, JAPAN

## Abstract

Myogenesis is regulated mainly by transcription factors known as Myogenic Regulatory Factors (MRFs), and the transcription is affected by epigenetic modifications. However, the epigenetic regulation of myogenesis is poorly understood. Here, we focused on the epigenomic modification enzyme, PHF2, which demethylates histone 3 lysine 9 dimethyl (H3K9me2) during myogenesis. *Phf2* mRNA was expressed during myogenesis, and PHF2 was localized in the nuclei of myoblasts and myotubes. We generated *Phf2* knockout C2C12 myoblasts using the CRISPR/Cas9 system and analyzed global transcriptional changes via RNA-sequencing. *Phf2* knockout (KO) cells 2 d post differentiation were subjected to RNA sequencing. Gene ontology (GO) analysis revealed that *Phf2* KO impaired the expression of the genes related to skeletal muscle fiber formation and muscle cell development. The expression levels of sarcomeric genes such as *Myhs* and *Mybpc2* were severely reduced in *Phf2* KO cells at 7 d post differentiation, and H3K9me2 modification of *Mybpc2*, *Mef2c* and *Myh7* was increased in *Phf2* KO cells at 4 d post differentiation. These findings suggest that PHF2 regulates sarcomeric gene expression via epigenetic modification.

## Introduction

Skeletal muscles are responsible for basic functions such as locomotion and metabolism [1]. Skeletal muscles are formed via myogenesis during development. Myogenic Regulatory Factors (MRFs, namely *Myf5*, *Myod*, *Mrf4*, and *Myog*) are the basic loop helix-type transcription factors that regulate each step of the myogenesis [2–4]. Myogenesis, which involves coordination between cell proliferation and differentiation, participates in complex gene expression regulatory networks that exert their functions primarily by regulating intercellular signaling and the expression of specific genes. Differentiated myotubes express high levels of sarcomeric genes such as members of the myosin heavy chains (MyHC) family. Myogenic gene expression is regulated by multiple factors including DNA methylation, histone modifications, chromatin remodeling, and non-coding RNAs [5].

The epigenome, comprising the chemical modifications of DNA and histone proteins, affects transcription and regulates myogenesis [6,7]. Histone 3 lysine 9 di- and tri- methylations (H3K9me2 and H3K9me3) are suppressive marks, and H3K9me is regulated by methyltransferases and demethylases. The H3K9 methyltransferase *Setdb1* is required for muscle stem cell expansion and suppresses terminal myoblast differentiation [8]. Suv39h1, another H3K9 methyltransferase, interacts with MyoD to act as a checkpoint between proliferation and differentiation [9]. G9a, another H3K9 methyltransferase, inhibits myogenesis via its methyltransferase activity [10]. Lysine-specific demethylase 1 (*Kdm1*; *Lsd1*; *Aof2*), which demethylates H3K4me1/2 and H3K9me1/2, induces myogenesis by forming a complex with MyoD and Mef2 [11]. Jmjd1c, a H3K9me2 demethylase, is required for MyoD expression, and monoubiquitination of Jmjd1c by Deltex2 inhibits its activity [12]. H3K9 methylation and demethylation are thus important in regulating myogenesis.

PHF2 comprises an N-terminal plant homeodomain (PHD), a Jumonji C-domain (JmjC), and a short coiled-coil region [13]. The PHD binds to H3K4me3, a marker of transcriptional activation, while JmjC of PHF2 demethylates H3K9me2, a marker of transcriptional repression. By binding to the bivalent histone markers H3K4me3 and H3K9me2, PHF2 modifies them and converts them into markers of active histone. *Phf2* physiologically regulates adipogenesis, chondrogenesis, non-alcoholic fatty liver disease (NAFLD) progression, and memory formation [14–17], while its role in skeletal muscles remains unclear.

To address this, we evaluated the function of *Phf2* in C2C12 myoblast myogenesis, hypothesizing that PHF2 participates in myogenesis by demethylating H3K9me2. We generated *Phf2* knockout (KO) C2C12 myoblasts using the CRISPR/Cas9 system, and evaluated *Phf2* function in myogenesis by evaluating RNA-sequencing-based global transcriptional changes. The loss of PHF2 in C2C12 myoblasts reduced the expression of genes that compose muscle sarcomere structure. These findings provide evidence for an important function of PHF2 in regulating gene expression.

## Materials and methods

### Cell culture

The C2C12 cell line was kindly provided by Dr. Shinichiro Hayashi. C2C12 myoblasts were maintained at 37°C with 5% CO_2_ in high-glucose Dulbecco’s Modified Eagle’s Medium (DMEM) (4.5 mg/mL) (D6429-500ML, Sigma-Aldrich, St. Louis, MO, USA) containing 10% fetal bovine serum (FBS) (10099–141, Thermo Fisher Scientific, Waltham, MA, USA) and 1% penicillin-streptomycin (PS) (26253–84, Nacalai Tesque, Kyoto, Japan). When the C2C12 myoblasts reached 80% confluence, they were seeded at 2 × 10^5^ cells per well in 8-well plates (167064, Thermo Fisher Scientific). Two days later, the medium was replaced with DMEM containing 2% horse serum (HS) (16050–122, Thermo Fisher Scientific) and 1% PS. The medium was changed every 2 d.

### Immunofluorescence staining

C2C12 myotubes at 0 d and 7 d post differentiation were fixed with 4% paraformaldehyde-phosphate buffer (09154–85, Nacalai Tesque) for 10 min and permeabilized with phosphate buffer saline (PBS) containing 1% Triton at room temperature. The fixed cells were washed three times with PBS at 5 min intervals. Blocking performed using PBS containing 1% Triton and 2% HS at room temperature for 30 min. The primary antibodies used for staining were anti-PHF2 antibody (3497S, CST, Boston, MA, USA, 1:100) and anti-Myosin Heavy Chain (MyHC) antibody (MF20 clone, MAB4470, R&D Systems, Minneapolis, MN, USA, 1:100), with incubation overnight at 4°C. The secondary antibody for MyHC was anti-mouse IgG 555 (A28180, Thermo Fisher Scientific, 1:1000), and the secondary antibody for PHF2 was anti-rabbit IgG 448 (A21206, Thermo Fisher Scientific, 1:1000), with incubation in the dark at room temperature for 1 h. The nuclei were stained with DAPI (340–07971, FUJIFILM Wako Pure Chemical, Osaka, Japan). Cells were visualized under a fluorescence microscope (BZ-X800, KEYENCE, Osaka, Japan) at 10× and 20× magnifications.

### Plasmid construction

Four distinct guide RNAs targeting mouse *Phf2* were designed using the CHOPCHOP website [18] and were inserted into the pGuide-it-ZsGreen1 vector (Z2601N, Takara, Shiga, Japan) according to the manufacturer’s instructions. The sequences of the inserted oligos were as follows; Phf2 guide RNA1 (gRNA) sense: CCG GCA TAA AGC GGG TAA CGT CGT; Phf2 gRNA1 antisense: AAA CAC GAC GTT ACC CGC TTT ATG; Phf2 gRNA2 sense: CCG GTC ACG TTG AGA ACA CGC TTG; Phf2 gRNA2 antisense: AAA CCA AGC GTG TTC TCA ACG TGA; Phf2 gRNA3 sense: CCG GAG CGT GGT ACC ATG TCC TCA; Phf2 gRNA3 antisense: AAA CTG AGG ACA TGG TAC CAC GCT; Phf2 gRNA4 sense: CCG GTC TTC GCC GAC CAG GTG GAC; Phf2 gRNA4 antisense: AAA CGT CCA CCT GGT CGG CGA AGA; NC gRNA sense: CCG GTG AGA CGA AAA ACG TCT CA; NC gRNA antisense: AAA CTG AGA CGT TTT TCG TCT CA. The Phf2 gRNA1, gRNA2, gRNA3 and gRNA4 target exon 1, exon 5, exon 6 and exon 7 of *Phf2* gene, respectively. All plasmid sequences were verified via sequencing.

### Generation of *Phf2* KO C2C12 myoblasts

Transfection of pGuide-it plasmids was performed using jetPRIME reagent (101000027, Polyplus, Illkirch-Graffenstaden, France), according to the manufacturer’s instructions. Briefly, C2C12 cells were seeded in DMEM with 10% FBS at 5 × 10^4^ cells/well in 6-well plates on the day before transfection. Four pGuide-it-ZsGreen plasmids (500 ng each) were pooled and transfected into the C2C12 cells; 24 h after transfection, cells expressing ZsGreen1 were sorted using a cell sorter (SH800, Sony, Tokyo, Japan). Sorted cells were subcultured for expansion for six days. Then the cells were stored at -80°C.

### Western blotting

PHF2 levels in *Phf2* KO myoblasts on the 6 d after transfection were determined using the Simple Western System Wes (Bio-Techne, Minneapolis, MN, USA) according to the manufacturer’s instructions. Protein lysates were diluted to 1.0 mg/ml, and the primary antibodies were β-Actin (AC004, ABclonal, Woburn, MA, USA, 1:50) and PHF2 (3497S, CST, 1:250). The secondary antibody for β-Actin was anti-mouse (GENA9310-1ML, Merck, Darmstadt, Germany, 1:100), and the secondary antibody for PHF2 was anti-rabbit (024–206, Bio-Techne).

### RNA extraction

Cryopreserved *Phf2* KO and mock cells were differentiated into myotubes, and total RNAs were extracted at 0, 2, 4, and 7 days post differentiation using ISOGEN Reagent according to the manufacturer’s instructions and was quantified using a Nanodrop 1000 Spectrophotometer (Thermo Fisher Scientific). RNA purity was assessed using the 260 nm/280 nm ratio.

### Quantitative Real-Time PCR Analysis (qRT-PCR)

Reverse transcription of total RNA (500 ng) was performed using the PrimeScript RT Master Mix (RR036A, Takara). qRT-PCR was performed using a final volume of 10 μl with Power SYBR Green PCR Master Mix (4367659, Thermo Fisher Scientific) according to the manufacturer’s instructions. qRT-PCR was conducted using the StepOne Real-Time PCR System (Applied Biosystems, Waltham, MA, USA). The primer sequences are listed in Table 1. *Actb* was used as an internal control.

**Table 1:** The sequence of the oligonucleotides for qPCR.

Gene name	Forward (5’- 3’)	Reverse (5’- 3’)
*Actb*	GGCTGTATTCCCCTCCATCG	CCAGTTGGTAACAATGCCATGT
*Myh7*	AGGGCGACCTCAACGAGAT	CAGCAGACTCTGGAGGCTCTT
*Myh2*	ACTTTGGCACTACGGGGAAAC	CAGCAGCATTTCGATCAGCTC
*Myh4*	GCTTGAAAACGAGGTGGAAA	CCTCCTCAGCCTGTCTCTTG
*Myh1*	TACTCACGCCAGCTAGACGA	TGCCTCTTCAGCTCCTCAAT
*Myod1*	TACCCAAGGTGGAGATCCTG	CATCATGCCATCAGAGCAGT
*Myog*	GAGACATCCCCCTATTTCTACCA	GCTCAGTCCGCTCATAGCC
*Mef2c*	GTCAGTTGGGAGCTTGCACTA	CGGTCTCTAGGAGGAGAAACA
*Mylk2*	GCGAGACAACAGACCTCGTC	GGTGTCCCCTTGCACCTTAG
*Mybpc2*	ATGCCTGAGGCTAAACCAGC	ACACAGAGTCCGGCTTTTTCA
*Myf5*	TGCCAGTTCTCCCCTTCTGA	AGGCTGCTACTCTTGGCTCA
*Tnnt2*	CAGAGGAGGCCAACGTAGAAG	CTCCATCGGGGATCTTGGGT

### RNA-sequencing

Total RNAs extracted from the C2C12 cells on day 2 after differentiation were used for RNA-seq. Libraries were prepared using the NEBNext Poly(A) mRNA Magnetic Isolation Module (for PolyA selection) (E7490, New England Biolabs, Ipswich, MA, USA) and total RNA using the NEBNext Ultra ll Directional RNA Library Prep Kit (E7760, New England Biolabs) according to the manufacturer’s instructions. Sequencing reads were performed using a NovaSeq 6000 device (Illumina, San Diego, CA, USA) with a NovaSeq 6000 S4 Reagent Kit v1.5 (Illumina) using paired-end reads of 150 bp.

The reads were aligned against the mm10 mouse genome using HISTAT2, and the count of reads per gene was obtained using StringTie. Statistical analysis of differentially expressed genes was performed using exactTest in edgeR after normalization. RNA-seq data were deposited in the Gene Expression Omnibus (accession GSE226618). Gene enrichment, KEGG pathway, and BP DIRECT analyses were performed using DAVID Bioinformatics Resources [19,20]. Principal component analysis (PCA) and hierarchically clustered heatmaps were analyzed using iDEP.96 [21] (http://bioinformatics.sdstate.edu/idep/).

### CUT&RUN-qPCR analysis

CUT&RUN-qPCR was performed using the CUT&RUN Assay Kit (86652, CST) according to the manufacturer’s instructions. The *Phf2* KO and mock cells 4 d post differentiation were subjected for CUT&RUN-qPCR analysis. The 100,000 cells were harvested per reaction and bound to activated Concanavalin A beads and permeabilized. The bead-cell complex was incubated overnight with either an anti-Histone H3K9me2 antibody (ab1220, Abcam, Cambridge, UK) or a control IgG antibody (CST) at 4°C. Cells were washed three times with digitonin buffer, and resuspended in 50 μl pAG/MNase for 1h at RT. DNA fragments were purified using the NucleoSpin Gel and PCR Clean-up (740609.50, Takara).

Quantitative PCR was performed with GeneAce SYBR qPCR Mix II (313–09423, NIPPON GENE, Tokyo, Japan). The primer sequences are listed in Table 2. Each Ct values were normalized to *Actb*.

**Table 2:** The primer sequence for qPCR.

Gene name	Forward (5’- 3’)	Reverse (5’- 3’)
*Actb*	TGTTACCAACTGGGACGACA	ACCTGGGTCATCTTTTCACG
*Mef2c*	TGAAATGAAGCAAGTGTGTGC	TCCAATCGGAAGTCTATCCA
*Mybpc2*	GTTCCCAAGACCCAGGATTT	GAAAGAGGGGAACTGGAACC
*Myh7*	GAACATGCCATGCCACAAC	CCCAGTCCCTAGCCAGATTT

### Statistical analysis

Results are expressed as mean ± SD. Statistical comparisons between two groups were performed using unpaired Student’s *t*-tests. One-way ANOVA was used to analyze differences between multiple groups.

## Results

### PHF2 expression in C2C12 cells

**Fig 1:**
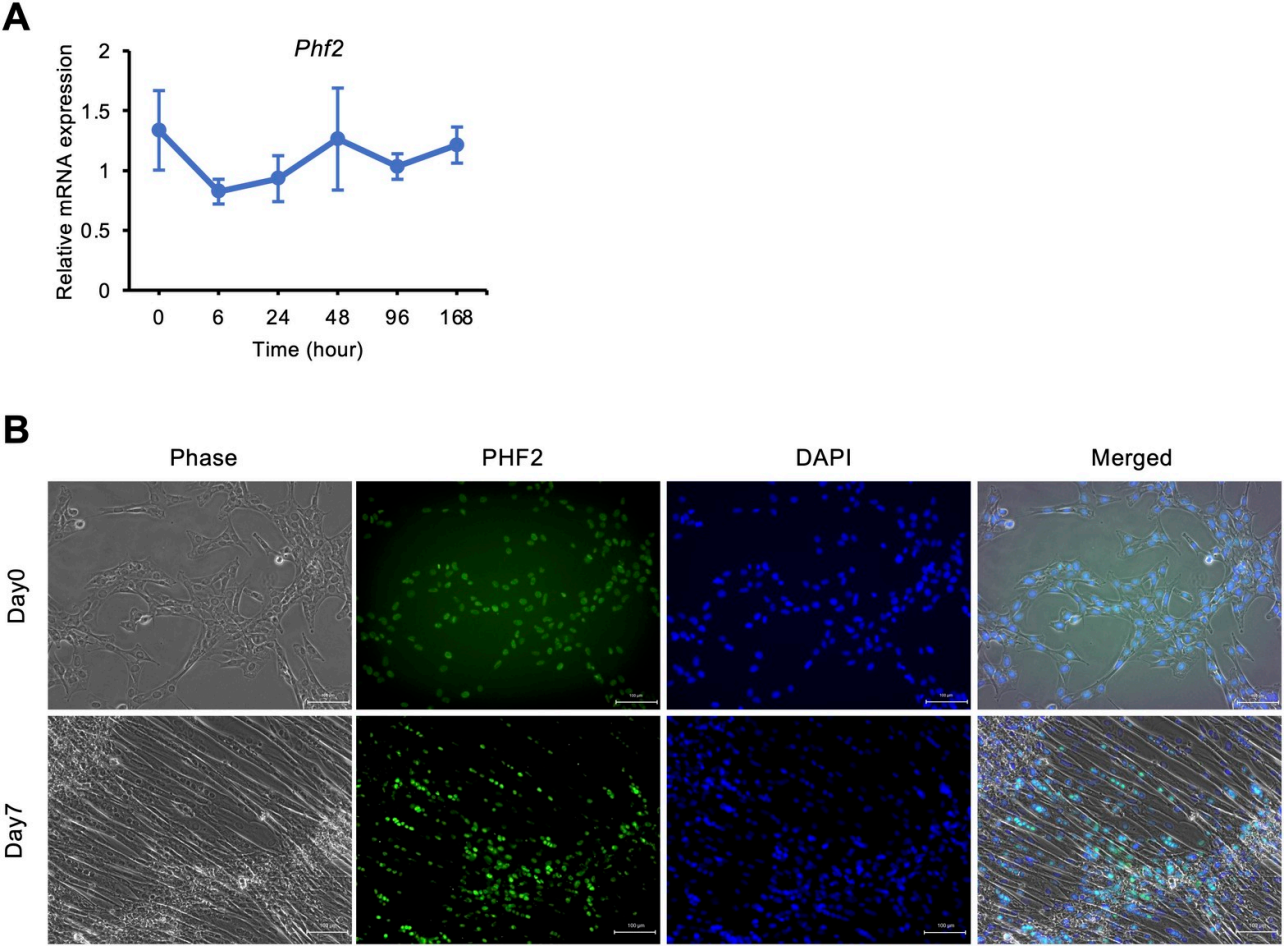
*Phf2* expression and PHF2 localization in C2C12 myotubes. (A) *Phf2* mRNA expression during differentiation in C2C12 myotubes (n = 4 wells per condition in one experiment). (B) Immunofluorescence staining of PHF2 (green) and DAPI (blue) in C2C12 myotubes on days 0 and 7 post differentiation. Scale bar, 100 μm.

We examined *Phf2* expression in C2C12 myotubes. *Phf2* mRNA expression during C2C12 myotube differentiation was determined (Fig 1A). *Phf2* was detected in C2C12 cells and its expression remained constant during differentiation. PHF2, a nuclear protein, is expressed in various organs [16,22]. Therefore, PHF2 localization was visualized via immunofluorescence. PHF2 was localized to the nuclei of C2C12 myoblasts and myotubes (Fig 1B). This PHF2 localization is consistent with its function as an H3K9me2 histone demethylase.

### Generation of *Phf2* KO C2C12 cells via CRISPR/Cas9

**Fig 2:**
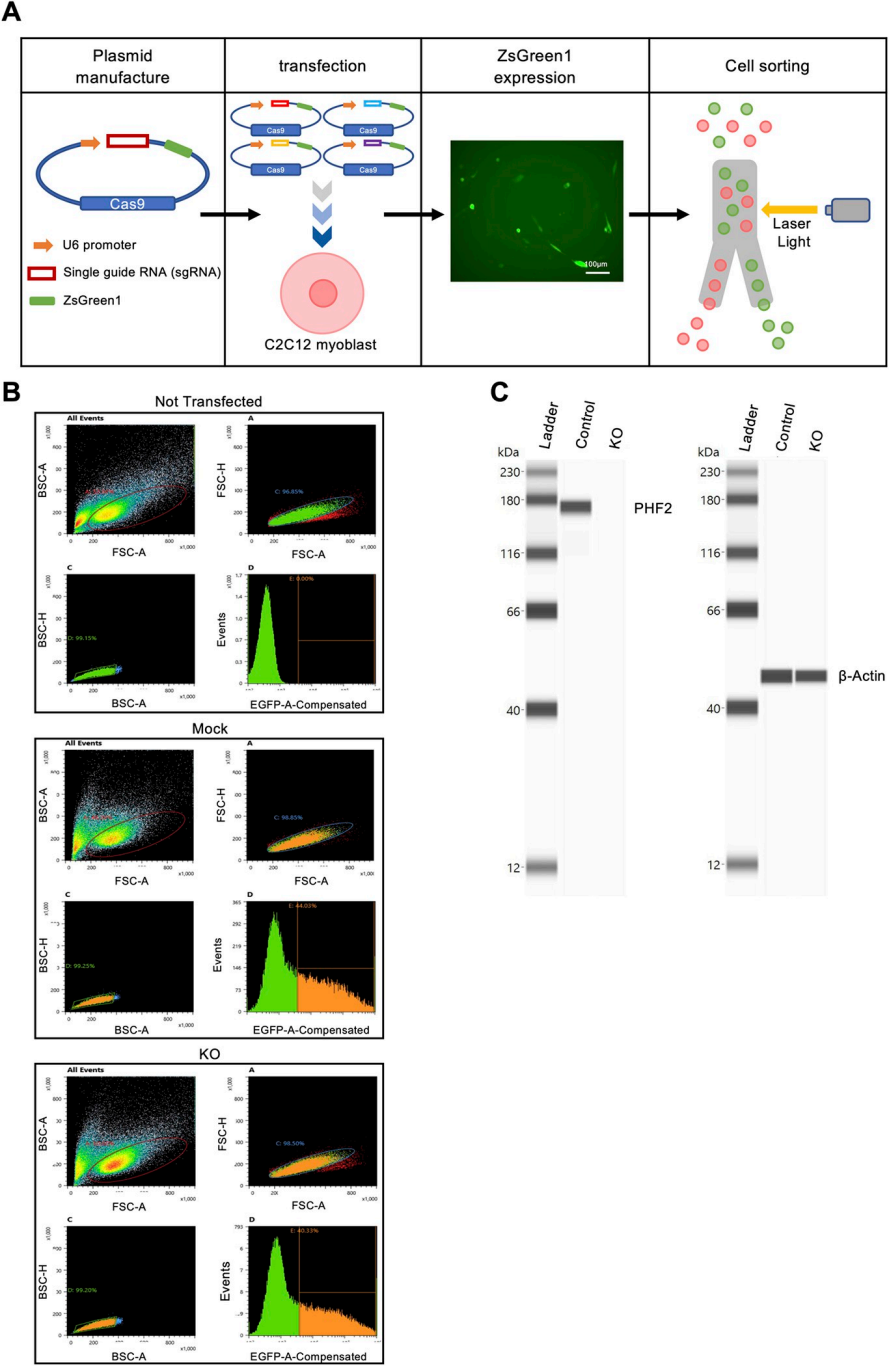
Generation of *Phf2* knockout (KO) cells in C2C12 myoblasts. (A) Overview of the generation of *Phf2* KO C2C12 cells. (B) Myoblasts expressing ZsGreen were obtained via cell sorting. Sorted untransfected C2C12 cells (without fluorescence) were used as negative controls. *Phf2* KO and mock cells were sorted under the same conditions. Sorted cells are indicated in the orange areas. (C) Protein levels of PHF2 were determined using the Simple Western System.

To clarify the function of *Phf2* in myogenesis, we generated *Phf2* KO C2C12 myoblasts. We designed four distinct *Phf2*-specific gRNA sequences using CHOPCHOP and constructed four plasmids containing the ZsGreen fluorescence reporter [18]. The four plasmids were pooled and transfected into C2C12 cells. As mock control, C2C12 cells transfected with a plasmid containing the ZsGreen fluorescence reporter without a specific gRNA was used. 24 hours after transfection, the cells were examined via fluorescence microscopy to confirm that the plasmid DNA was incorporated into the C2C12 myoblasts. Transfected cells were collected using a cell sorter (Fig 2A and 2B). PHF2 protein levels, determined using the Simple Western System, were 96.5% lower following *Phf2* knockout (Fig 2C).

### Differentiation of *Phf2* KO C2C12 cells

**Fig 3:**
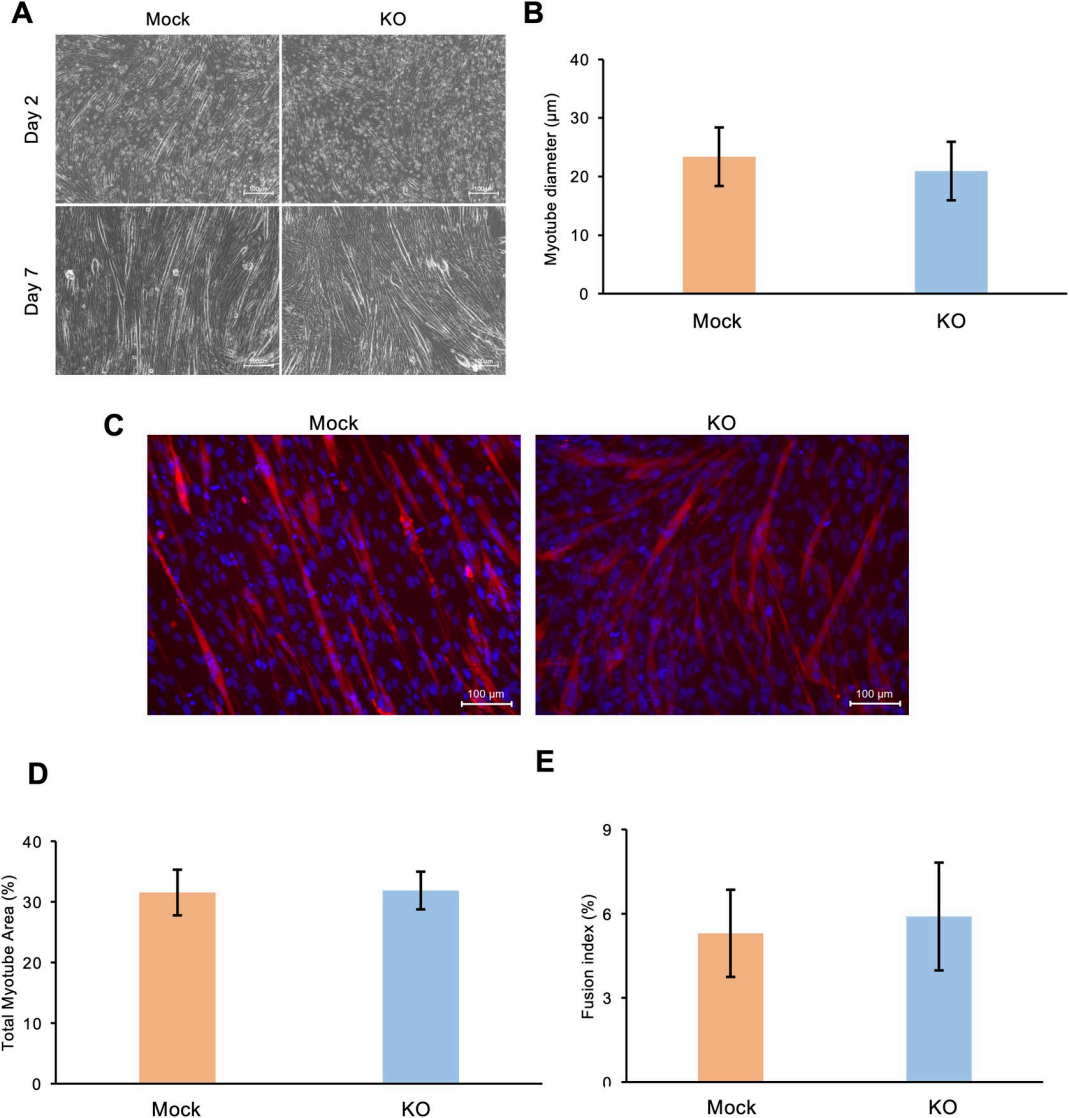
The effect of *Phf2* knockout (KO) on myotube formation at 7 d post differentiation. (A) Phase-contrast microscopy images of mock and *Phf2* KO cells 7 d post differentiation. Scale bar, 100 μm. (B) Myotube diameter in mock and *Phf2* KO cells 7 d post differentiation (n = 100 cells per group in one experiment). (C) Immunofluorescence staining of Myosin Heavy Chain (MyHC, red) and nuclei (blue). (D) Total MyHC-expressing area (n = 8, cells per group in one experiment). (E) Myotube fusion index based on the ratio of MyHC-nuclei to total nuclei (n = 10, cells per group in one experiment).

To analyze the myotube-formation capacity of *Phf2* KO cells, they were cultured for 7 d in the differentiation medium (Fig 3A). The myotube diameter in the *Phf2* KO group after 7 d differentiation was not significantly different from that of the mock group (Fig 3B). The differentiation status of *Phf2* KO cells 7 d after induction of differentiation was analyzed by immunofluorescence staining of MyHC (Fig 3C). *Phf2* KO did not affect total myotube area or myotube fusion index (Fig 3D and 3E). These results suggest that *Phf2* KO did not affect C2C12 myotube formation.

### RNA-seq analysis of *Phf2* KO C2C12 cells

**Fig 4:**
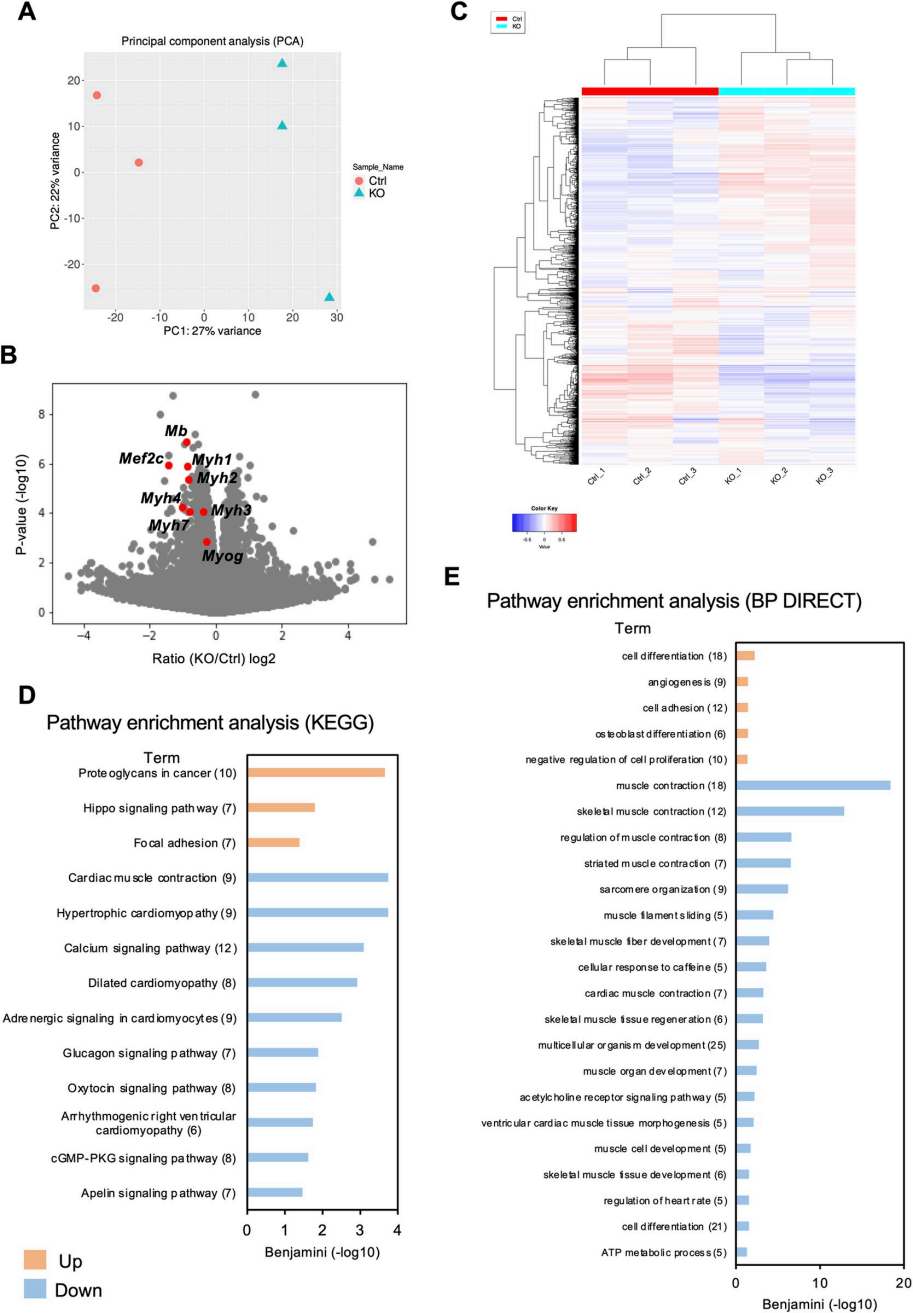
RNA-sequence analysis of *Phf2* knockout (KO) cells at 2 d post differentiation. (A) Principal components analysis with RNA-seq analysis of *Phf2* control cells and *Phf2* KO cells (n = 3 wells in each group in one experiment). (B) Volcano plot of the RNA-seq data obtained from *Phf2* KO cells 2 d post differentiation. (C) Heatmap visualizing the expression profiles based on RNA-seq analysis of *Phf2* control and *Phf2* KO cells (n = 3 wells per group). (D) KEGG pathway analysis of differentially expressed genes (DEGs) in *Phf2* KO C2C12 cells. Numbers in parentheses indicate the number of genes associated with each enriched term. (E) BP DIRECT analysis of DEGs in *Phf2* KO C2C12 cells. Numbers in parentheses indicate the number of genes associated with each enriched term.

To analyze transcription in *Phf2* KO cells, RNA sequencing was performed on *Phf2* KO C2C12 cells 2 d post differentiation. In total, 279 significantly differentially expressed genes (DEGs) were identified in *Phf2* KO cells (*q* < 0.05), with 102 upregulated and 177 downregulated (Fig 4A–4C). Interestingly, the expression of genes involved in myogenesis and muscle differentiation, such as *Mef2c* and *Myog*, was lower in the *Phf2* KO cells than in the WT cells (Fig 4B). KEGG and BP DIRECT analyses were used to examine the biological functions of genes with significantly different expression [23]. KEGG pathway analysis of the 102 upregulated and 177 downregulated genes in *Phf2* KO revealed that the downregulated pathways included cardiac muscle contraction (*Atp2a1/1a2*, *Cacna1s*, *Casq2*, *Hrc*, *Myh6/7*, *Myl4*, *Tnnt2*), calcium signaling (*Atp2a1*, *Cacna1s*, *Camk2a*, *Casq2*, *Erbb3*, *Fgfr1*, *Hrc*, *Pdgfb*, *Ryr1/3*, *Tnnc2*, *Tpcn1*), and adrenergic signaling in cardiomyocytes (*Atp2a1/1a2*, *Cacna1s*, *Camk2a*, *Myh6/7*, *Myl4*, *Akt2*, *Tnnt2*) (Fig 4D). BP DIRECT analysis revealed enrichment by the downregulated genes of muscle contraction (*Actn2*, *Cacna1s*, *Chrnb1*, *Lmod3*, *Myom3*, *Myh1/2/3/4/6/7/8*, *Myl1/6b*, *Ryr1*, *Tnni1*, *Tnnt1/2*), skeletal muscle fiber development (*Stac3*, *Cacna1s*, *Klhl40*, *Lmod3*, *Myh4*, *Plec*, *Ryr1*), skeletal muscle tissue regeneration (*Klf5*, *Dysf*, *Dag1*, *Mymk*, *Mymx*, *Plau*), and muscle-cell development (*Actn2*, *Cacna1s*, *Chrnb1*, *Dysf*, *Neb*) (Fig 4E). Focal/cell adhesion was enriched by the upregulated genes (Fig 4D and 4E). Gene Ontology analysis revealed that PHF2-targeted genes were mainly associated with muscle contraction, muscle cell development, and skeletal muscle fiber development.

### PHF2 regulates the expression of genes involved in muscle terminal differentiation via demethylating repressive H3K9me2 mark

We validated the RNA-seq results via qPCR and determined the expression patterns of the main genes in myogenesis. *Myod1*, *Myf5*, and *Myog* are myogenic regulators of transcription factors that induce myotube formation [24–26]. qRT-PCR revealed that *Myod1* and *Myog* were upregulated in the mock cells 2 d post differentiation, and that *Myod1*, *Myf5*, and *Myog* expression was slightly reduced in *Phf2* KO cells, however, the expression levels were not changed at 7 d post differentiation (S1 Fig). Considering the results of myotube formation (Fig 3), the reduction of *Myod1* and *Myog* at 2 d post differentiation is not physiologically important in early myogenesis.

**Fig 5:**
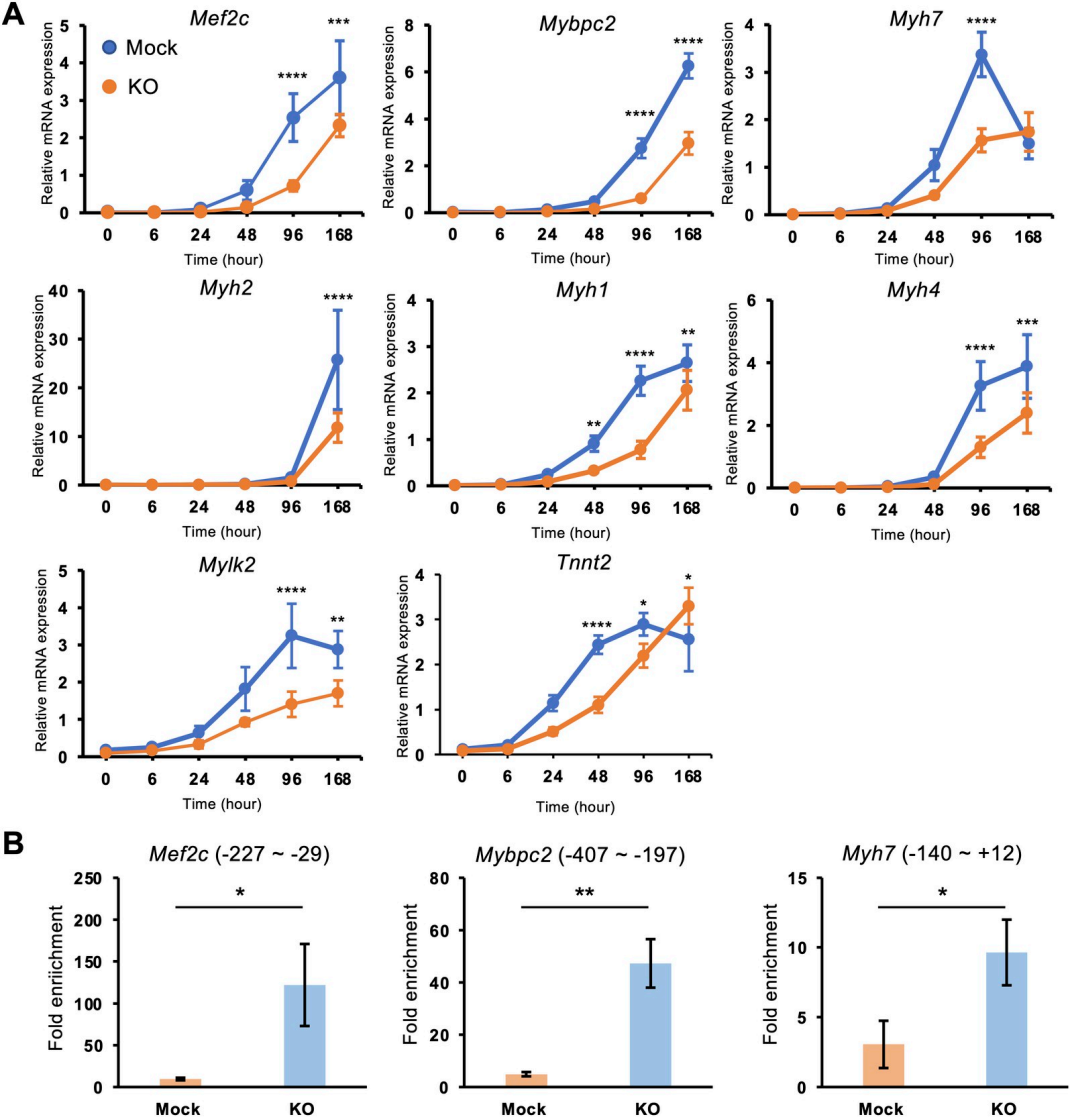
Expression of genes and recruitment of H3K9me2 related to muscle myogenesis in *Phf2* KO cells. **(A)** mRNA expression of *Mef2c*, *Mybpc2*, *Myh7*, *Myh2*, *Myh1*, *Myh4*, *Mylk2*, and *Tnnt2* in mock and *Phf2* KO cells based on qRT-PCR (n = 4 wells per condition in one experiment). (B) CUT&RUN-qPCR analysis of H3K9me2 mark in the promoter region of *Mef2c*, *Mybpc2*, and *Myh7* using mock and *Phf2*-KO cells (n = 3 wells per condition in one experiment). The amplicon positions from TSS were indicated in parentheses. *P < 0.05, **P < 0.01, ***P < 0.001, and ****P < 0.0001 compared with mock C2C12 myotubes.

Then we analyzed the expressions of terminal differentiation genes (*Mef2c*, *Myh*s, and *Tnnt2*) that are highly expressed in differentiated myotube and mainly encode muscle sarcomeres. We also included *Mylk2* and *Mybpc2* which were not highly expressed at 48 h post differentiation but were highly elevated on 7 d post differentiation. Their induction was significantly impaired in *Phf2* KO myotubes (Fig 5A). These results indicated that *Phf2* regulated the expression of sarcomeric genes highly expressed at terminal differentiation.

As PHF2 is known to act as an epigenetic activator by removing H3K9me2 [22,27,28], we performed CUT&RUN-qPCR assay with a H3K9me2 antibody using *Phf2* KO and mock cells on 4 d post differentiation. *Phf2* KO myotubes increased the H3K9me2 mark at the promoter region of *Mef2c*, *Mybpc2*, and *Myh7* (Fig 5B). Taken together, these findings indicate that PHF2 plays an important role in inducing sarcomeric genes via demethylating H3K9me2 during myogenesis.

## Discussion

In this study, we generated *Phf2* KO cells using the CRISPR/Cas9 system and analyzed their transcriptional function during myogenesis via RNA sequencing. This revealed that PHF2 is required for the expression of sarcomeric genes such as *Myh*s and *Mybpc2*.

The CRISPR/Cas9 system was used to generate KO cells for the genes of interest [29–31]. Using the CRISPR/Cas9 system to obtain KO cells, single-cell cloning is required, owing to the heterogeneity of mutations. However, repeated passage can easily result in the loss of myogenic function in C2C12 cells. Therefore, single-cell cloning was not performed after sorting. Instead, to increase the knockout efficiency, we developed four distinct gRNAs and transfected the pooled plasmid (Fig 2). The *Phf2* KO cells remained viable even after cryopreservation. The KO cell generation method used here could be useful for the KO of other cells with differentiation capacities, such as 3T3-L1 or MC3T3-E1 cells.

Three H3K9 methyltransferases (*Setdb1*, *Suv39h1*, and *G9a*) and two H3K9 demethylase (*Lsd1 and Jmjd1c)* have been reported to regulate myogenesis; *Setdb1*, *Suv39h1*, and *G9a* inhibited myogenesis [8–10], while *Lsd1 and Jmjd1c* accelerate it [11,12]. A complex mechanism involving three methyltransferases and two demethylases is involved in H3K9 methylation during muscle differentiation. Here, we found that *Phf2*, also an H3K9 demethylase, positively regulated sarcomeric gene expression during myogenesis (Fig 5B). Therefore, our results are consistent with previous reports on H3K9 methylation during myogenesis, and we proposed another regulator of myogenesis.

These findings reveal that *Phf2* KO impaired the expression of genes involved in myogenesis (*Myog* and *Myod1)* and in sarcomeric processes (*Myhs* and *Mybpc2*) (Figs 4 and 5). However, myotube formation (in terms of myotube diameter, total myotube area, and fusion index) did not differ significantly between *Phf2* KO and mock cells (Fig 3). Conversely, suppression of sarcomeric genes may affect myofunctions such as muscle contraction, energy metabolism, and fiber-type changes. Maximal fast-twitch muscle strength is indeed suppressed in *Mybpc2* mutant mice [32].

Additionally, it remains unclear whether these genes are direct targets of PHF2. To answer these questions, future ChIP-seq experiments and physiological analyses of muscle function in PHF2 deficient mice are necessary. Systemic *Phf2* KO mice exhibit approximately 70% mortality within 3 d of birth, with reduced adipose tissue mass in the surviving mice [14]. The generation of skeletal muscle-specific *Phf2* KO mice is essential to further elucidate the function of PHF2 in skeletal muscles.

### Limitations of the study

In this study, we used the mock control as a control of *Phf2* KO cells. The mock cells were introduced the control plasmid containing Cas9, ZsGreen, and gRNA. However, the gRNA sequence of the control plasmid does not match the mouse genomic sequence. Therefore, the double-strand break would not happen in the mock control cells. The effect of double-strand break itself on C2C12 transcription is not excluded in this study. In addition, C2C12 cells produce immature myotubes compared to skeletal muscle in *vivo*, therefore, the present study could not clarify the role of *Phf2* in sarcomere formation and muscle contractility.

## Supporting information

S1 Fig Expression levels of genes involved in muscle myogenesis in *Phf2* KO. (TIF)
