# Supplementary figures and images for: PHF2 regulates sarcomeric gene transcription in myogenesis
Source: PLoS One. 2024 May 3;19(5):e0301690. doi: 10.1371/journal.pone.0301690 (PMC11068198; doi:10.1371/journal.pone.0301690)

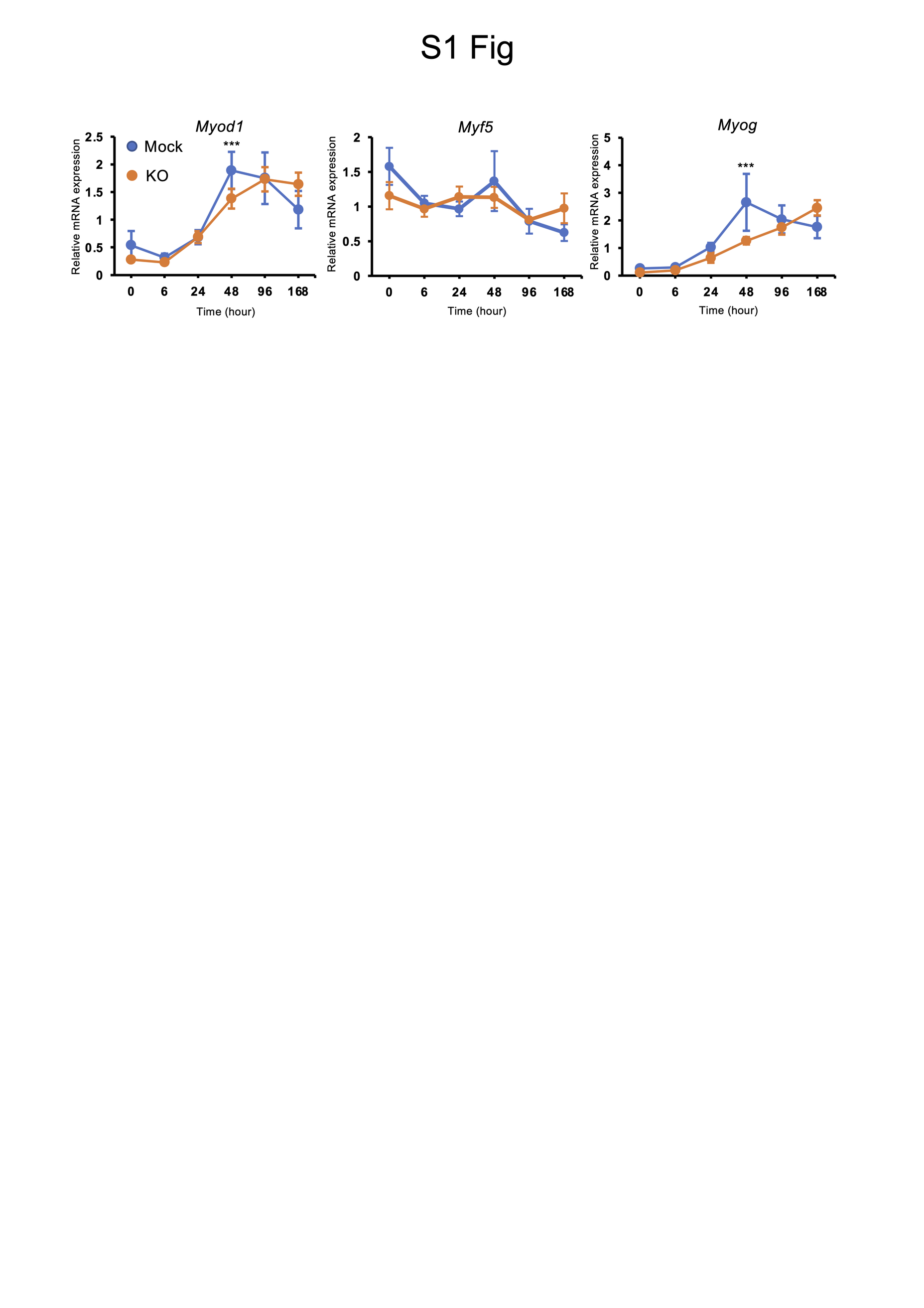

Supplement: S1 Fig — (TIF) [file pone.0301690.s001.tif]
